# The journal of diabetes is adopting open access

**DOI:** 10.1111/1753-0407.13221

**Published:** 2021-09-16

**Authors:** Zachary Bloomgarden, Guang Ning

**Affiliations:** ^1^ Department of Medicine, Division of Endocrinology, Diabetes, and Bone Disease Icahn School of Medicine at Mount Sinai New York New York USA; ^2^ Academician of the Chinese Academy of Engineering, Director of Shanghai Clinical Center for Endocrine and Metabolic Disease, Director of Shanghai Institute for Endocrinology and Metabolism President of Ruijin Hospital affiliated Shanghai JiaoTong University School of Medicine

The 2020 Impact Factor for the *Journal of Diabetes*, announced by Clarivate on 30 June 2020, reached the level of 4.006. The impact factor is calculated by dividing the number of citations in 2020 by the number of articles published during the prior 2 years. Thus, as an approximation, a given article in the journal will have been cited four times. This is not of course the only way in which manuscripts published by the journal become visible, and we constantly strive to help accepted articles reach more of our audience of researchers and practitioners involved in the understanding and treatment of diabetes. Our Table of Contents includes authors' capsule summaries and graphic elements to show aspects of each manuscript, and for many years we have made at least one accepted article, as well as our monthly commentaries, freely downloadable.

The Editors and Associate Editors of the journal have looked with great interest at the growth of the Open Access model of medical publishing. Fundamentally, we consider highly desirable the goal of making the research and commentary of the journal fully available to all those involved in the understanding and treatment of diabetes. In a sense, Open Access began in the medical sciences in mid‐1997, when Medline (the Medical Literature Analysis and Retrieval System Online) was made available via PubMed, a bibliographic database of life sciences and biomedical information^1^ used by most of those in the field. For those of us whose professional childhood began with trips to the medical library to pore over monthly and yearly printed (and weighty) tomes of *Index Medicus*, PubMed was nothing short of revolutionary, and we recall the excitement with which we greeted its initial appearance. With Open Access, particularly as more journals move to this model, authors can count on reaching an audience far larger than that of a subscription‐based journal, essentially all of those with internet access, allowing greater visibility and impact of their work. With a click on the Table of Contents, and soon with a click on PubMed Central (https://www.ncbi.nlm.nih.gov/pmc/), readers will have access to the full details of all articles we publish rather than just having access to abstracts and one table or figure. This should translate into greater support of a more sustainable model for the work of education carried out by libraries and universities.^2^ As with many other journals in the field,^3^ we are now committed to a move to Open Access to begin in the first issue of 2022. The dilemma, and one about which we have had a great deal of internal debate, is that the cost of carrying out the activities of the journal, with peer‐review processes and maintenance of high publishing standards, will no longer be funded by subscriptions. We have wrestled with the unavoidable manuscript charges that will be required under Open Access. Arguments have been made on both sides as to whether this is or is not a desirable development.^4^ We fully expect that charges will usually be borne by employers or funders and in certain cases partially or fully waived (and we encourage authors to apply for this when their financial capacity is limited). But the benefits will include greater visibility as more readers will be able to access published articles, and we will continue to endeavor to process manuscripts rapidly to help with their more widespread dissemination.

As we complete our 13th year of continuous publication we invite you, our readers and collaborators, to continue to participate in what has been a collaboration of researchers from throughout the world, representing a dialogue between East and West via all aspects of epidemiology, etiology, pathogenesis, management, complications and prevention of diabetes, including the molecular, biochemical, and physiological aspects of diabetes. We celebrate our accomplishments and look to the future.
